# Transient Neurological Deficit Due to Intracerebral Hemorrhage: A Diagnostic Pitfall Mimicking a Transient Ischemic Attack

**DOI:** 10.7759/cureus.85355

**Published:** 2025-06-04

**Authors:** Indika Wettasinghe, Asanka Ratnayake, Suresh Mendis

**Affiliations:** 1 Faculty of Medical Sciences, University of Sri Jayewardenepura, Colombo, LKA; 2 Department of Internal Medicine, Colombo South Teaching Hospital, Colombo, LKA

**Keywords:** intracerebral hemorrhage, neurology, stroke, transient ischemic attack, transient neurological weakness

## Abstract

Intracerebral hemorrhage (ICH) is a severe and potentially fatal subtype of stroke that typically presents with persistent or worsening neurological deficits. In contrast, transient neurological deficits are commonly attributed to transient ischemic attacks (TIAs). As a result, many clinicians initiate antiplatelet therapy without prior brain imaging, especially in settings with limited resources. However, this approach may lead to misdiagnosis and inappropriate treatment in rare cases where ICH mimics a TIA.

A 64-year-old man with a history of hypertension presented with sudden-onset left-sided weakness and slurred speech lasting one hour, which resolved spontaneously. He had no history of trauma, substance use, or antithrombotic therapy. On admission, neurological deficits were improving, with limb power returning to normal within 15 minutes. Initial clinical assessment suggested a transient ischemic attack (TIA), but a non-contrast computed tomography (CT) scan of the brain revealed a small intracerebral hemorrhage in the right basal ganglia.

This case highlights the diagnostic challenge of distinguishing TIA from ICH based solely on clinical presentation. Although uncommon, ICH can occasionally manifest with transient neurological symptoms, closely mimicking ischemic events. In such scenarios, initiating antithrombotic therapy without imaging carries the potential risk of hematoma expansion. Recommendations regarding brain imaging in suspected TIA differ across expert bodies, while some advocate early neuroimaging in all patients presenting with transient neurological symptoms, others recommend a more selective approach based on the clinical suspicion of alternative diagnoses. These differences may reflect variations in healthcare systems and underscore the importance of applying clinical judgment.

This case underscores the importance of considering ICH in the differential diagnosis of transient neurological symptoms. It advocates for early brain imaging, preferably before starting antiplatelet therapy, in all patients with suspected TIA, to ensure safe and appropriate management. Greater clinical vigilance and imaging access are essential to prevent misdiagnosis and improve outcomes in such atypical presentations.

## Introduction

Intracerebral hemorrhage (ICH) is defined as bleeding into the brain parenchyma, usually resulting from the rupture of small penetrating blood vessels and not caused by trauma [1]. It is a severe form of stroke that is associated with significant morbidity and a high risk of mortality [2]. Timely identification and appropriate management are essential to improve outcomes in these patients [3]. Generally, ICH presents with a sudden onset of neurological deficits that often worsens over time. Although transient symptoms have been noted in patients with subdural hematomas and subarachnoid hemorrhages caused by amyloid angiopathy [4,5], transient neurological deficits resembling transient ischemic attacks (TIAs) have seldom been reported in patients with ICH [6].

TIA is defined as a transient episode of neurological dysfunction caused by focal brain, spinal cord, or retinal ischemia, without evidence of acute infarction on neuroimaging [7]. The term “transient” typically refers to symptoms resolving within 24 hours, although many resolve within minutes. Antiplatelet therapy is indicated following a TIA to reduce the risk of recurrent ischemic stroke, unless contraindicated, as the risk of recurrence is highest in the days immediately following the initial event [8].

The differential diagnosis of transient neurological deficits includes ischemic events (TIA or minor stroke) and a range of stroke mimics, including seizures, migraines, metabolic disturbances, and functional neurological disorders [7,9]. Among these, ICH is often not initially suspected in transient presentations due to its perceived association with persistent or worsening symptoms. This diagnostic uncertainty is further compounded by guideline recommendations and clinical practice, especially in resource-limited settings where antiplatelets may be initiated prior to brain imaging and access to computed tomography (CT) scanning is limited or unavailable. This approach may inadvertently lead to the premature administration of antiplatelet therapy in patients with unrecognized ICH, posing a potential risk of hematoma expansion and clinical deterioration [10].

Here, we present a case where a patient presenting with a transient weakness and slurring of speech was found to have an ICH instead of a TIA or minor stroke. This case highlights the potential for ICH to present with transient deficits and emphasizes the need for careful clinical assessment and timely imaging to avoid misdiagnosis and inappropriate treatment.

## Case presentation

A 64-year-old man with a history of hypertension presented with a sudden onset of left-sided upper limb and lower limb weakness and slurred speech for one hour, which then resolved spontaneously. According to the patient’s history, there was complete weakness of the limbs at onset, which improved considerably by the time he reached the hospital. There was no history suggestive of head trauma, smoking, or substance abuse. The patient had a 10-year history of hypertension and had been on losartan 50 mg twice daily, though with poor follow-up and suboptimal blood pressure control. He was not taking any other medications, notably antiplatelets or anticoagulants. There was no past medical history or family history of stroke, transient ischemic attacks, or ischemic heart disease.

On admission, slurring of speech was noted, and the power of left upper and lower limbs was 4/5, which improved to 5/5 within 15 minutes. The pulse rate was 78 beats per minute with a regular rhythm, and the blood pressure was 190/100 mmHg. The rest of the cardiovascular, respiratory, and abdominal examinations were normal. Based on the history and examination, the patient was initially diagnosed with a transient ischemic attack (TIA).

**Figure 1:**
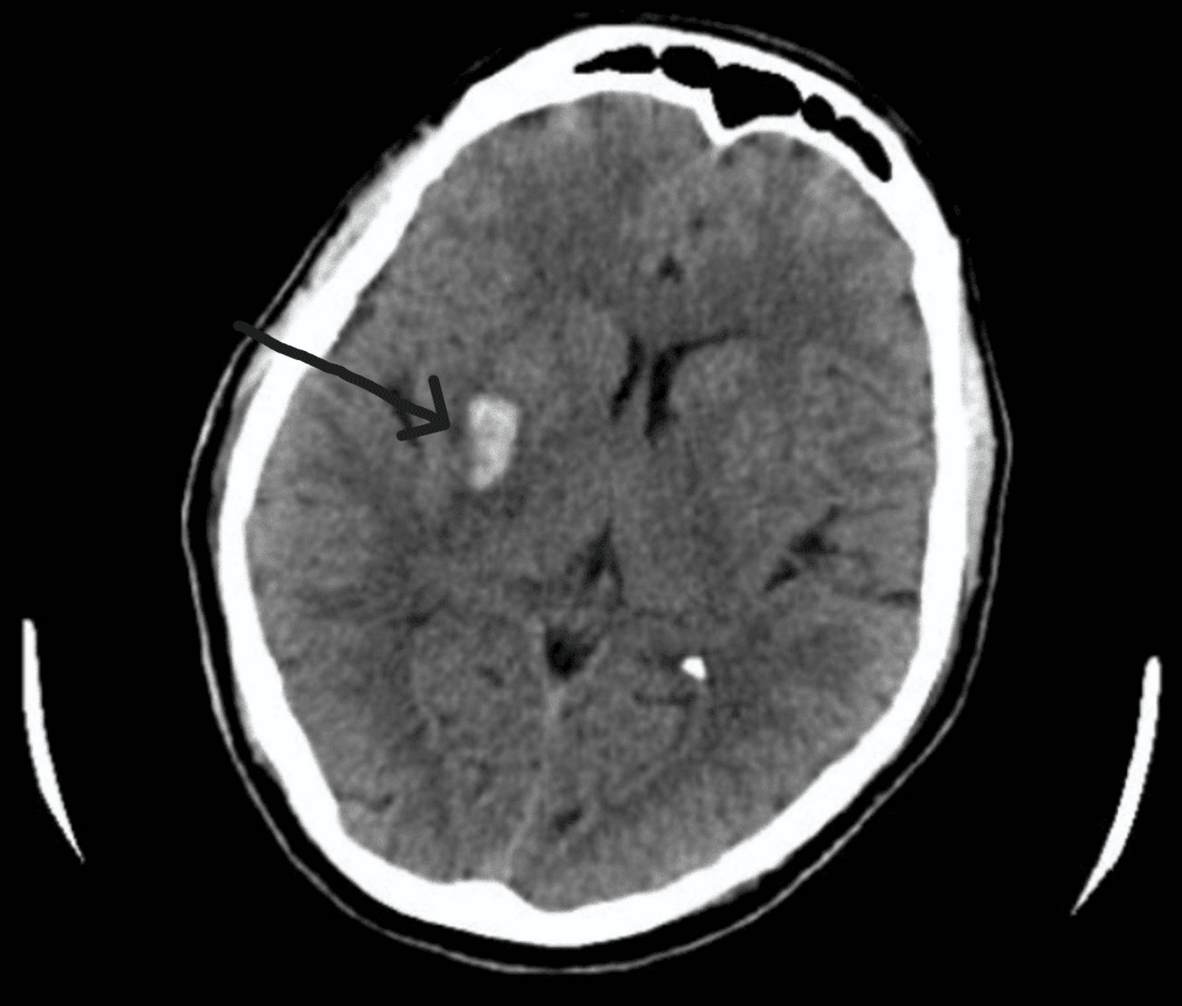
Non-contrast CT of the brain showing a right-sided intracerebral hemorrhage in the basal ganglia region, with minimal surrounding mass effect. CT: computed tomography

However, a non-contrast computed tomography (CT) scan of the brain revealed a small ICH in the right basal ganglia with minimal mass effect (Figure 1). The patient was given intravenous labetalol 10 mg stat, followed by oral antihypertensives, and the neurological status was monitored. The systolic blood pressure was maintained at 130-140 mmHg. The initial laboratory tests, including full blood count, coagulation profile, and liver and renal function tests, were within normal limits, and the ECG was in sinus rhythm.

Over the next 24 hours, there was complete resolution of his slurred speech. A repeat CT scan showed no significant changes in the size or location of the ICH. The 2D echocardiogram showed left ventricular hypertrophy, with no regional wall motion abnormalities and a preserved ejection fraction. The patient was discharged with a plan for outpatient rehabilitation. Due to resource limitations, advanced imaging such as magnetic resonance imaging (MRI) was not available in our setting. Given the patient’s clinical improvement and the limited availability of CT, a repeat scan was not performed, as it was not expected to alter clinical management.

## Discussion

Several studies have documented neurological deterioration after an ICH, but very few studies have reported rapid improvement in neurological deficits in patients with ICH [6,11]. Notably, Kumar et al. [6] and Gunatilake [12] have noted that, to their knowledge, no prior studies had described such rapid reversibility in clinical deficits among patients with ICH [13]. In their 14-year retrospective study, Kumar et al. identified 2,137 patients with spontaneous ICH, excluding those with subarachnoid or subdural hemorrhage, hemorrhagic infarction, or tumor-related bleeding. Among these, they identified a unique subgroup of 17 patients whose neurological deficits resolved within 24 hours [6].

The mechanism by which transient deficits occur in ICH is not fully understood but may involve temporary perihematomal edema, spreading cortical depression, or transient dysfunction caused by mass effect or the irritation of adjacent cortical or subcortical structures. Kumar et al. hypothesized that symptom resolution may result from the rapid dissipation of edema or the redistribution of blood without causing permanent tissue injury [6]. Another hypothesis includes seizure-related postictal deficits or transient ischemia in the setting of small hematomas causing localized pressure effects [6,11]. Literature has also shown that small hemorrhage size and location (e.g., putamen) may allow rapid symptom resolution due to limited tissue destruction [12]. Furthermore, while not classified as typical ICH, lobar hemorrhages due to cerebral amyloid angiopathy (CAA) can be preceded by transient focal neurological episodes (TFNEs), which are brief spells of weakness or sensory changes that mimic TIA [6,14].

Our case report shows that transient neurological symptoms due to ICH can mimic a TIA and highlights the need to obtain urgent brain imaging in every patient with a transient neurological weakness, especially before starting antiplatelets.

This is of significant clinical relevance as studies indicate that in many settings, patients presenting with transient neurological weakness are often not admitted to the emergency department as a standard practice [15]. In one study, 10% of patients eventually diagnosed with ICH had been given antiplatelet therapy before imaging was performed, though no immediate harm was reported [6]. Nonetheless, the theoretical risk of hematoma expansion or worsening hemorrhage remains a serious concern, especially in elderly patients or those with underlying hypertension. Gunatilake notes that clinicians tend to diagnose transient ischemic attacks on symptoms alone and to start antiplatelet drug treatment pending the results of computed tomography, especially in developing countries where computed tomography is scarce [12]. This practice carries not only safety concerns but also important medicolegal implications.

Current guidelines may inadvertently reinforce this gap. The 2019 National Institute for Health and Care Excellence (NICE) guideline recommends not offering CT of the brain imaging to patients with suspected TIA unless there is clinical suspicion of an alternative diagnosis and advises starting aspirin 300 mg daily immediately unless contraindicated [16]. This recommendation is based on the low prevalence of ICH among true TIA presentations and the benefits of early antiplatelet therapy in preventing recurrent ischemic events. However, our case and others like it suggest that rare but serious mimics such as ICH may be overlooked under this approach.

In contrast, the American Heart Association/American Stroke Association (AHA/ASA) guidelines recommend performing non-contrast CT or MRI (class I) in all patients with transient neurological symptoms to distinguish ischemia from hemorrhage and stroke mimics [17]. MRI with diffusion-weighted imaging (DWI) is particularly useful when available, as it detects acute ischemic lesions and microbleeds that influence treatment decisions [17]. The divergence between the NICE and AHA/ASA guidelines may reflect differences in healthcare system structures, evidence interpretation, and clinical priorities. The NICE guidelines are shaped by the United Kingdom’s publicly funded healthcare model, emphasizing cost-effectiveness and resource allocation. Consequently, they place greater weight on evidence suggesting that immediate imaging in typical TIA presentations has limited impact on outcomes. In contrast, the AHA/ASA guidelines align with a healthcare system that offers broader access to diagnostic modalities and prioritizes the role of imaging in detecting atypical presentations, such as hemorrhage or stroke mimics. While the UK approach favors rapid clinical assessment and the early initiation of antiplatelets to prevent recurrence, the US model emphasizes diagnostic precision through imaging to guide individualized treatment.

Chronic hypertension is the most common underlying cause of spontaneous intracerebral hemorrhage (ICH), particularly in deep brain structures such as the basal ganglia, thalamus, pons, and cerebellum [1,18]. The basal ganglia are especially vulnerable due to the presence of small penetrating arteries, such as the lenticulostriate branches of the middle cerebral artery, which are exposed to continuous high-pressure flow in hypertensive states. Over time, this leads to lipohyalinosis, fibrinoid necrosis, and microaneurysm formation (Charcot-Bouchard aneurysms), predisposing the vessel walls to rupture [1,19,20]. These pathophysiological changes explain why the basal ganglia are one of the most frequent sites of hypertensive ICH. Neuroimaging and autopsy studies consistently demonstrate a high prevalence of deep ICH in hypertensive individuals [21].

## Conclusions

This case report underscores the potential for misdiagnosing intracerebral hemorrhage (ICH) as a transient ischemic attack (TIA) in patients presenting with transient neurological symptoms, a clinical pitfall that may result in the inappropriate use of antiplatelets and delays in definitive management. It highlights the value of early neuroimaging in all cases of transient neurological deficits to ensure accurate diagnosis. As demonstrated here, timely and appropriate intervention can significantly improve patient outcomes. Ultimately, stroke care should be patient-centered, with decisions guided by the clinical context, individual risk profile, and resource availability.
